# Disseminated Syringomas of the Upper Extremities in a Young Woman

**DOI:** 10.7759/cureus.3619

**Published:** 2018-11-21

**Authors:** Kavina Patel, Ashley D Lundgren, Ammar M Ahmed, Anthony C Soldano

**Affiliations:** 1 Dermatology, University of Texas Health Science Center, San Antonio, USA; 2 Dermatology, Dell Medical School - The University of Texas, Austin, USA; 3 Dermatology, Clinical Pathology Associates, Austin, USA

**Keywords:** acral syringoma, upper extremity, eruptive, papule, eccrine gland

## Abstract

Syringomas are benign, eccrine sweat gland tumors frequently found on the eyelids and neck in post-pubescent women and may present in healthy individuals or be associated with various medical comorbidities. We present a case of an otherwise healthy 19-year-old female with an abrupt onset of disseminated syringomas on the bilateral forearms and dorsal hands. Eruptive acral syringomas have not been previously reported in adolescents, and this diagnosis should be considered in patients presenting with a papular eruption on the hands and forearms.

## Introduction

Syringomas are benign, adnexal tumors of unknown etiology that derive from eccrine sweat ducts [1]. They are most commonly located bilaterally on the inferior eyelids of post-pubescent women and present as multiple, white to yellow, 1 to 3-millimeter papules; on occasion, they are found in alternate locations such as the cheeks, axillae, abdomen, chest, and groin [2]. A clear cell variant, where cells have colorless cytoplasm due to glycogen build-up, is commonly associated with diabetes mellitus [3]. Eruptive syringomas are a less common presentation and may surface abruptly in adolescents, typically on the abdomen. Syringomas have increased incidence in patients with Down syndrome, those with Asian heritage, and in women [2]. Hormone levels are postulated to play a role in driving syringoma development given the increased incidence in women, with proliferation also noted during pregnancy, puberty, and the premenstrual period [4]. There are also reports of eruptive syringomas on medications such as carbamazepine and antiepileptic drugs. Syringomas are not known to have hereditary transmission but some familial occurrences have been reported [5]. Friedman and Butler developed four classifications of syringomas based on clinical features: localized, familial, eruptive, and a form associated with Down’s syndrome [6]. A healthy 19-year-old woman presented with eruptive acral syringomas—the first recorded case of this condition in an adolescent to our knowledge.

## Case presentation

**Figure 1:**
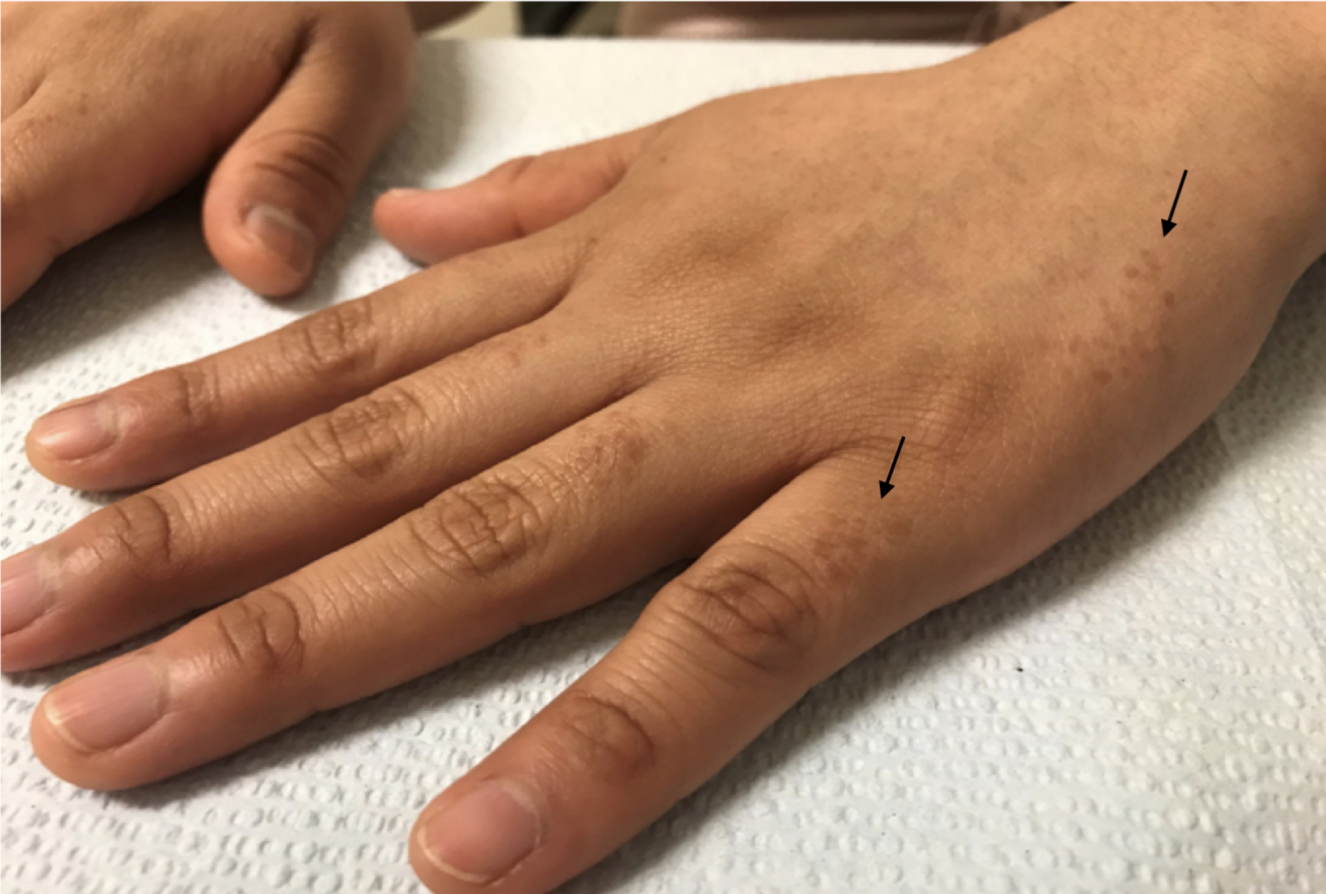
Light brown, monomorphic ovoid papules symmetrically distributed on the interphalangeal skin (left arrow) and lateral left hand (right arrow)

**Figure 2:**
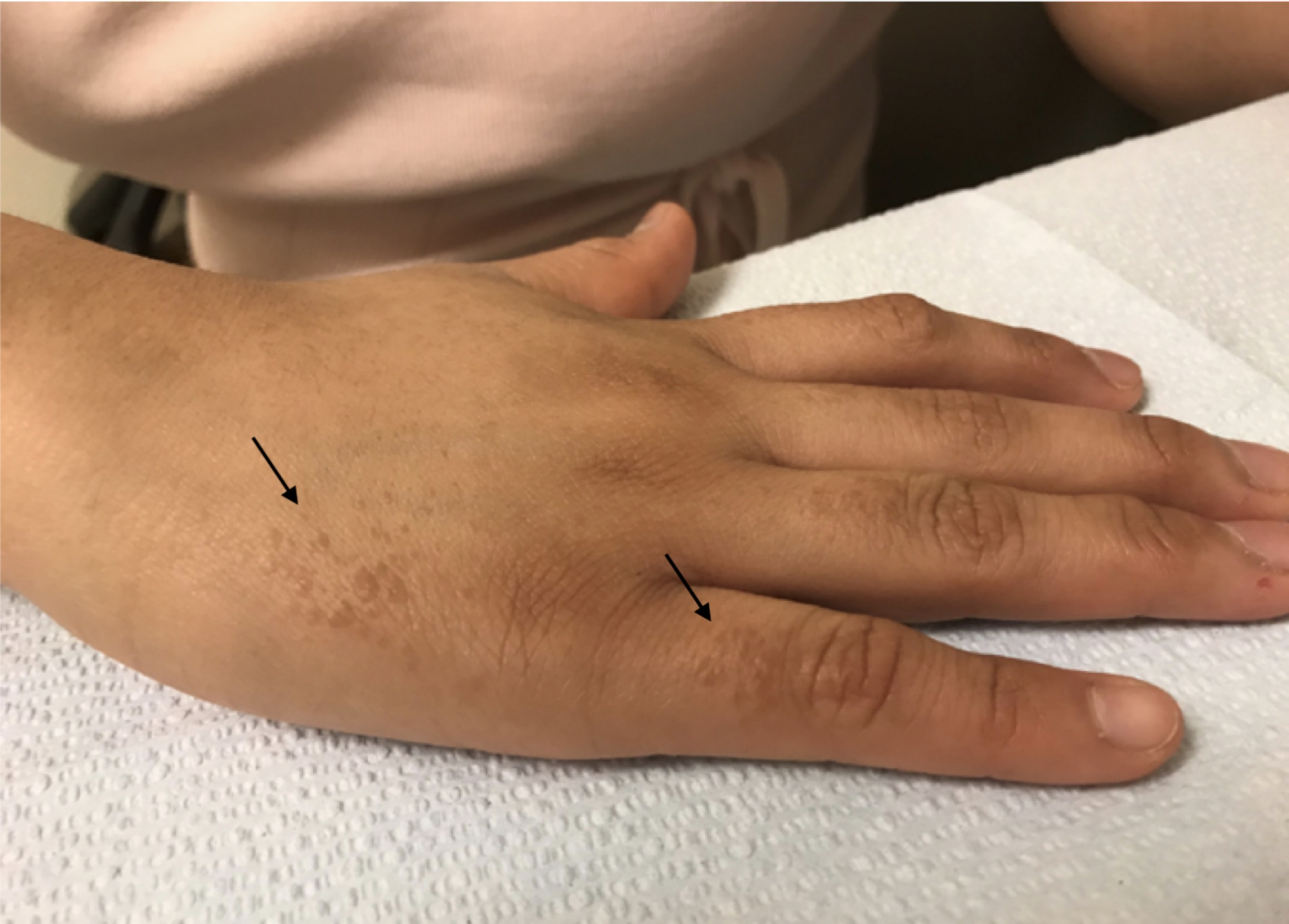
Light brown, monomorphic ovoid papules symmetrically distributed on the interphalangeal skin (right arrow) and lateral right hand (left arrow)

**Figure 3:**
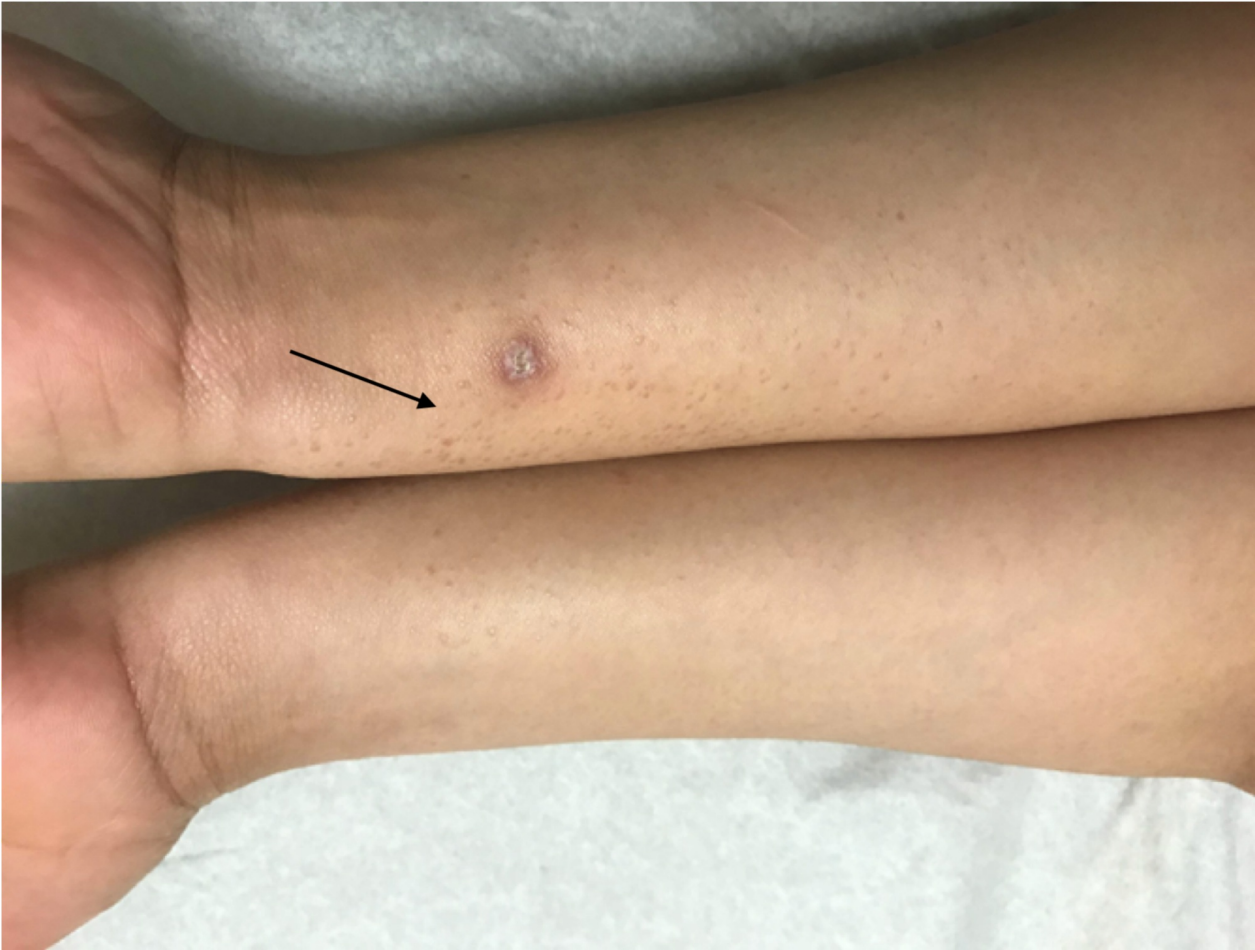
Light brown ovoid papules (arrow) symmetrically distributed on the flexor forearms

**Figure 4:**
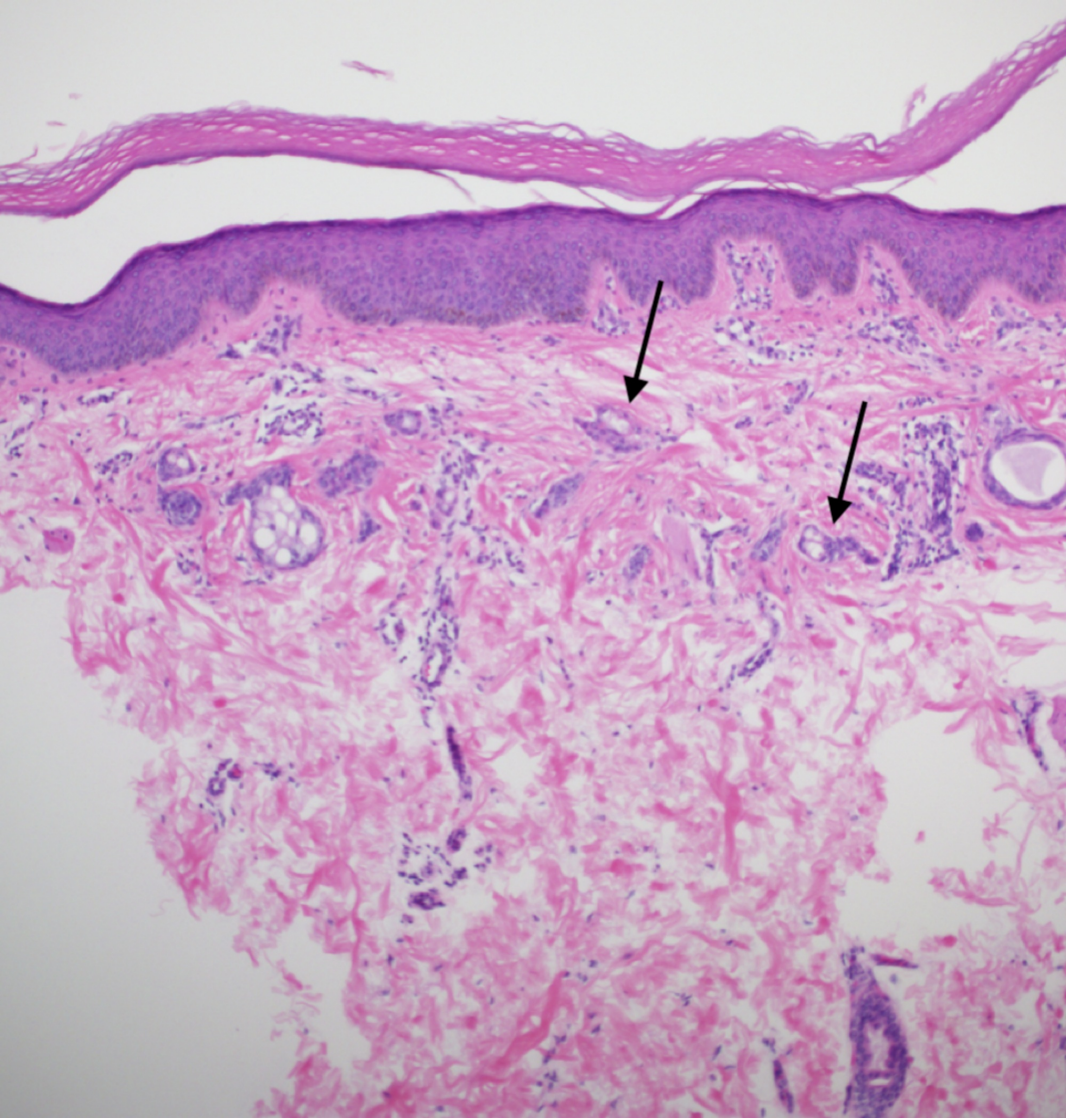
Hematoxylin and eosin (H&E) 10x view of numerous small ducts (arrows) resembling tadpoles embedded in a fibrous stroma. The walls of the ducts are lined by two rows of epithelial cells. The deeper dermis is uninvolved

A 19-year-old Hispanic female presented with complaints of an eruption of the hands and forearms that had started one year prior. She reported mild associated pruritus, which increased with exposure to natural sunlight. She denied the involvement of the head, trunk, lower extremities, or genitalia. She took no daily medications and had no chronic medical problems. There was no evidence of conditions associated with syringomas on history, physical exam, or workup. The physical exam revealed numerous, light brown, ovoid papules on the dorsal hands and fingers and on the dorsal and ventral surfaces of the forearms, with some areas of confluence on the lateral dorsal hands (Figures 1-3). Similar lesions were not observed elsewhere, including the groin, on the patient. A punch biopsy was obtained from the right forearm to establish the diagnosis and revealed a proliferation of small eccrine ductal structures lined by cuboidal cells within a fibrous stroma with an unremarkable epidermis, consistent with a diagnosis of syringoma. No cytologic atypia or significant infiltration of the deeper dermis by these ductal structures was appreciated (Figure 4). A limited laboratory analysis was conducted and revealed a normal complete blood count, as well as a glycated hemoglobin test of 5.2% (normal < 5.7%).

## Discussion

To our knowledge, our case appears to be the first report of eruptive acral syringomas in an adolescent. Eruptive acral syringomas are extraordinarily rare with a total of 13 previous cases reported in the English-language medical literature (Table 1) [7]. Of these, the average patient age was 48 years, with the youngest report occurring in a 27-year-old and nine of the 13 were in females. The majority of these cases documented no associated disease, although several were associated with malignancy, including carcinoid tumor, melanoma, breast cancer, and promyelocytic leukemia. All 13 cases had an involvement of the upper extremities, and three cases also included involvement in the infraorbital and periorbital regions and the breast. The incidence of acral syringoma may be underreported since these lesions are generally small and asymptomatic and often go unnoticed by the patient and physician [3].

**Table 1:** Previous cases of acral syringomas

Year	Sex	Age	Location	Associated condition	Author
1977	Male	31	Dorsal hands	None	Hughes and Apisarnthanarax [8]
1982	Female	35	Dorsal hands and infraorbital	None	Asai [9]
1982	Male	52	Forearms and dorsal wrist	Clear cell acanthoma	Van den Broek [10]
1989	Male	52	Ventral forearms	Carcinoid tumor	Berbis [11]
1990	Male	69	Ventral forearms and dorsal hands	Basal cell carcinoma	Metze [12]
1997	Female	43	Dorsal hands and feet	Breast cancer	Garcia [13]
1998	Male	43	Forearms and wrist	None	Patrizi [14]
1998	Female	60	Forearms and breast scar	Breast tubular adenoma	Patrizi [14]
2006	Female	43	Forearms	Photosensitivity reaction	Martin-Garcia [15]
2008	Female	28	Dorsal hands	None	Muniesa [16]
2009	Female	27	Dorsal phalanges	None	Koh [17]
2009	Female	41	Ventral forearms	Periorbital trichoepitheliomas	Balci [18]
2009	Female	44	Posterior forearms	None	Valdivieso-Ramos [19]
2015	Female	62	Ventral forearms and periorbital	Promyelocytic leukemia	Varas-Meis and Prada [7]
2018	Female	19	Dorsal hands and forearms	None	Patel, Lundgren, Ahmed, Soldano (present study)

Lesional specimens will typically exhibit a “paisley-tie” pattern in the superficial to mid-dermis, with tadpole-shaped ducts embedded in a red, sclerotic stroma and will exhibit positive staining against keratin, carcinoembryonic antigen, and S-100 proteins [3]. A differential diagnosis for the patient based on the clinical examination included hyperpigmented flat warts, lichen planus, atypical polymorphous light eruption, adnexal neoplasms, and acrokeratosis verruciformis of Hopf. Syringomas do not require treatment unless there are cosmetic or symptomatic concerns, and interventions include laser therapy, excision, cryotherapy, dermabrasion, topical atropine, and topical tretinoin, which have been used with variable results [4,7]. The diagnosis and treatment options were discussed with the patient, and she declined medical or surgical intervention. She remains in good health without the development of additional lesions in the six subsequent months following her initial presentation. Based on the short follow-up, there is a possibility that additional lesions will appear in other areas, particularly the groin in a female patient.

## Conclusions

Our case demonstrates a unique example of a healthy, 19-year-old female with eruptive syringomas on the upper extremities. This diagnosis should be entertained in patients presenting with a papular eruption on the hands and forearms.
